# Arrhythmogenic Interaction Between Sympathetic Tone and Mechanical Stretch in Rat Pulmonary Vein Myocardium

**DOI:** 10.3389/fphys.2020.00237

**Published:** 2020-03-26

**Authors:** Yuriy V. Egorov, Leonid V. Rosenshtraukh, Alexey V. Glukhov

**Affiliations:** ^1^Laboratory of Heart Electrophysiology, Russian Cardiology Research Centre, Institute of Experimental Cardiology, Moscow, Russia; ^2^Department of Medicine, School of Medicine and Public Health, University of Wisconsin–Madison, Madison, WI, United States

**Keywords:** pulmonary veins, stretch, arrhythmia, mechano-electrical response, adrenalin

## Abstract

Rapid firing from pulmonary veins (PVs) frequently initiates atrial fibrillation, which is a common comorbidity associated with hypertension, heart failure, and valvular disease, i.e., conditions that pathologically increase cardiomyocyte stretch. Autonomic tone plays a crucial role in PV arrhythmogenesis, while its interplay with myocardium stretch remains uncertain. Two-microelectrode technique was used to characterize electrophysiological response of Wistar rat PV to adrenaline at baseline and under mild (150 mg of applied weight that corresponds to a pulmonary venous pressure of 1 mmHg) and moderate (10 g, ∼26 mmHg) stretch. Low concentrations of adrenaline (25–100 nmol/L) depolarized the resting membrane potential selectively within distal PV (by 26 ± 2 mV at baseline, by 18 ± 1 mV at 150 mg, *P* < 0.001, and by 5.9 ± 1.1 mV at 10 g, *P* < 0.01) suppressing action potential amplitude and resulting in intra-PV conduction dissociation and rare episodes of spontaneous activity (arrhythmia index of 0.4 ± 0.2, NS vs. no activity at baseline). In contrast, 1–10 μmol/L of adrenaline recovered intra-PV propagation. While mild stretch did not affect PV electrophysiology at baseline, moderate stretch depolarized the resting potential within distal PV (-56 ± 2 mV vs. -82 ± 1 mV at baseline, *P* < 0.01), facilitated the triggering of rapid PV firing by adrenaline (arrhythmia index: 4.4 ± 0.2 vs. 1.3 ± 0.4 in unstretched, *P* < 0.001, and 1.7 ± 0.8 in mildly stretched preparations, *P* < 0.005, at 10 μmol/L adrenaline) and induced frequent episodes of potentially arrhythmogenic atrial “echo” extra beats. Our findings demonstrate complex interactions between the sympathetic tone and mechanical stretch in the development of arrhythmogenic activity within PVs that may impact an increased atrial fibrillation vulnerability in patients with elevated blood pressure.

## Introduction

Focal electrical activity from pulmonary veins (PVs) frequently initiates atrial fibrillation (Haissaguerre et al., 1998; Chen et al., 1999), which is a common comorbidity associated with hypertension, heart failure, and valvular disease (Schotten et al., 2011). These conditions cause hemodynamic atrial overload resulting in pathologically increased cardiomyocyte stretch and facilitates the arrhythmogenic activity from PVs (Tsao et al., 2001). In the setting of atrial dilatation, mechanoelectrical feedback has been linked to the development of ectopic beats that trigger atrial fibrillation. However, the precise mechanisms remain poorly understood.

We (Egorov et al., 2015, 2019) and others (Arora et al., 2003; Walters et al., 2014; Pasqualin et al., 2018) have demonstrated a significant electrophysiological heterogeneity of myocardial cells within PVs, which may form a functional substrate for focal activity and echo extra beats. This was linked to a distinct ionic channel and Ca^2+^-handling gene repertoire in PVs that would underlie their distinct response to proarrhythmic stimuli. Our recent study revealed a tension-dependent stretch-induced depolarization of the resting membrane potential (RP) within the distal part of rat PV, which decreased action potential (AP) amplitude (APA) and triggered conduction discontinuities and both ectopic and reentrant activities within the vein (Egorov et al., 2019). One may suggest that such stretch-induced arrhythmogenesis would significantly interfere with autonomic tone that plays a crucial role in triggering rapid firing from PVs (Patterson et al., 2005; Doisne et al., 2009). It was shown that the differences in the RP and reaction to adrenergic stimulation between the PV and the left atrium leads to automatic electrical activity occurring specifically in PV (Doisne et al., 2009). Indeed, PV myocytes demonstrated a low density of the K_ir_2. x channels and the RP stabilizing inwardly rectifying current *I*_K1_ (Melnyk et al., 2005; Tsuneoka et al., 2017), increased resting Na^+^ permeability (Malecot et al., 2015), and enhanced chloride conductance (Okamoto et al., 2014).

In this study, we hypothesized that myocardial stretch would facilitate the development of arrhythmogenic ectopic activity induced by sympathetic stimulation in the PV. To test this, two-microelectrode technique was used to characterize a region-specific electrophysiological response of rat PV myocardium to low (25–100 nmol/L) and high (1–10 μmol/L) concentrations of adrenaline at baseline and under mild (150 mg of applied weight, which corresponds to a PV pressure of 1 mmHg) and moderate (10 g, ∼26 mmHg) stretch.

## Materials and Methods

### Animals and Preparations

All methods and protocols used in these studies have been approved by the Animal Care and Use Committee of the Cardiology Research Center (Moscow, Russia) following the Guidelines for Care and Use of Laboratory Animals published by the National Institutes of Health (NIH) (publication no. 85-23, revised 1996). All animals used in this study received humane care in compliance with the Guide for the Care and Use of Laboratory Animals. Adult (8- to 12-month-old) Wistar rats (*n* = 7) of both sexes were used. Rats were anesthetized with urethane 2 g/kg with heparin (0.2 U), and the loss of pain reflex was evaluated to assure the appropriate level of anesthesia. The isolated PV preparation was performed as described previously (Egorov et al., 2015, 2019). Briefly, after mid-sternal incision, the heart with lungs was removed and placed in oxygenated (95% O_2_, 5% CO_2_) room-temperature Tyrode solution of the following composition (in mmol/L): 118 NaCl, 1.8 CaCl_2_, 1.2 MgCl_2_, 4.7 KCl, 1.2 NaH_2_PO_4_, 25 NaHCO_3_, and 11 glucose (pH = 7.35 ± 0.05). The left atrium (LA) together with the LA appendage and PV region was dissected from the ventricles, right atrium, and interatrial septum. The preparation then was placed in a tissue bath (2.5 ml) and continuously superfused with oxygenated Tyrode’s solution (18 ml/min) at 37 ± 0.5°C. Central PV was cleaned from fat and lung tissues, beside the LA, lanced and then positioned on a thin coat of silicon on the bottom of a tissue bath with the endocardial side facing upward. The pacing electrode was placed on the edge of the left atrial appendage. A small portion of the end−distal part of the PV was not cut open and was used to weave a silk suture (4−0) with a weight applied.

### Microelectrode Recordings

**FIGURE 1:**
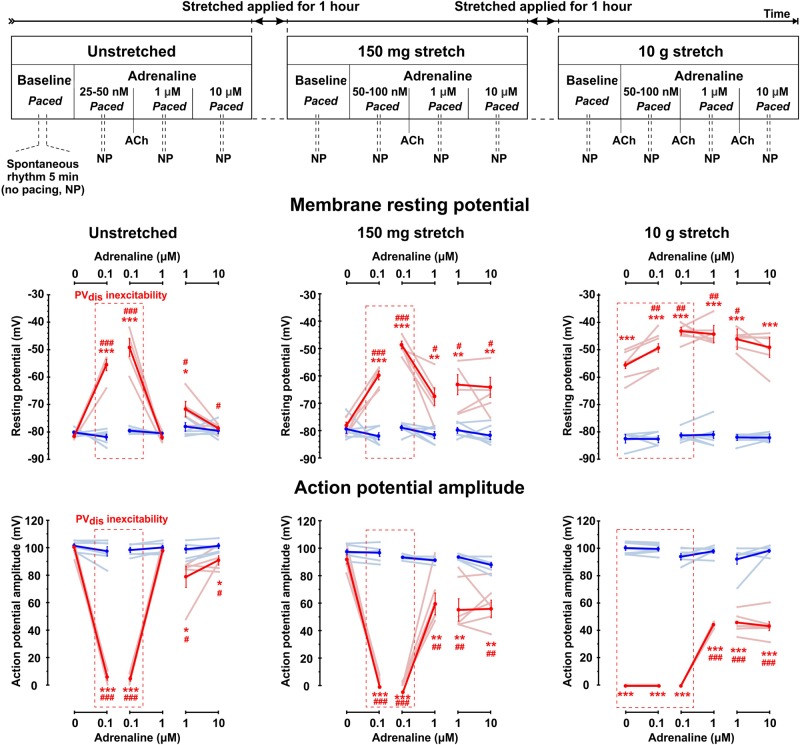
Complex interactions between sympathetic tone and mechanical stretch in rat pulmonary vein (PV) myocardium. **Top:** Experimental protocol for testing different levels of sympathetic stimulation in unstretched and stretched PV preparations. Physiological status of PV_dis_ inexcitability induced be either low concentrations of adrenaline (25–100 nmol/L), or pathological stretch (10.5 g) was tested by a brief application of acetylcholine (ACh), which was administered to hyperpolarize the RP and recover PV_dis_ excitability and then washed out to continue experimental protocol. All measurements were performed during constant left atrial pacing, except brief (5–10 min) periods when pacing was stopped (no pacing, NP), and spontaneous rhythm was recorded if present. Below, changes in the resting membrane potential (middle panels) and action potential amplitude (low panels) are shown for distal (red traces) and ostium (blue traces) PV regions at baseline (no adrenaline applied) and under low (25–100 nmol/L) and high (1–10 μmol/L) concentrations of adrenaline measured in unstretched and stretched (150 mg and 10 g) preparations. Changes are shown for individual rats (light blue and light red lines) as well as mean ± SEM (solid lines). Red dashed box indicates the presence of PV_dis_ inexcitability. *N* = 6–7 rats. *P < 0.05, **P < 0.01, ***P < 0.001 for PV_dis_ vs. PV_ost_; ^#^*P* < 0.05, ^##^*P* < 0.01, ^###^*P* < 0.001 within the same group vs. baseline by repeated measurements two-way ANOVA with Bonferroni correction.

Transmembrane potentials were simultaneously recorded from the endocardial surface of the distal (PV_dis_) and the ostial (PV_ost_) parts of the PV (Egorov et al., 2019) using two glass microelectrodes filled with 3.0 mmol/L KCl (tip resistance, ∼10–40 MΩ) and connected to high-input impedance amplifiers (WPI model KS-701, World Precision Instruments, New Haven, CT, United States). Microelectrodes were stably maintained within the tissue during all the measurements within each experimental condition (unstretched, 150 mg and stretch, 10 g; Figure 1). In some preparations, microelectrode stability was lost during 1-h stretch applications (i.e., between experimental conditions), and repenetration in nearby tissue was performed. Transmembrane potential signals were recorded, digitized (sampling rate of 5 kHz) using analog-digital converter (E-154, L-Card, Moscow, Russia), and then saved on a computer for offline analysis as described previously (Egorov et al., 2015). To characterize electrophysiological properties of the PV myocardium, RP and APA were measured during S1S1 = 300 ms pacing. The pacing current was at least 2× the pacing threshold.

### Experimental Protocol

**FIGURE 2:**
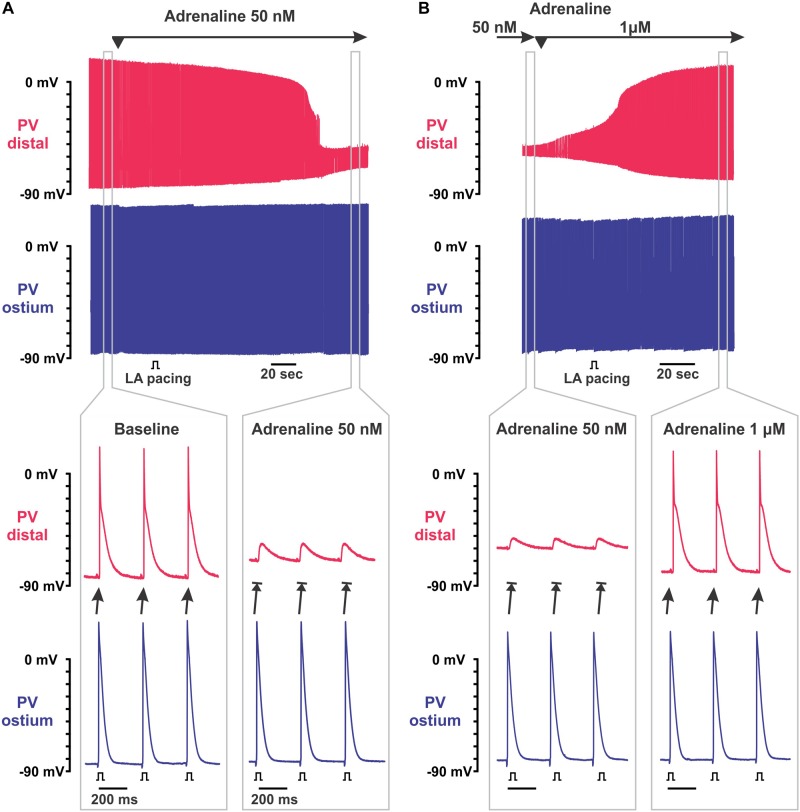
Biphasic effect of adrenaline on intra-pulmonary vein (PV) conduction. **(A)** Application of 50 nmol/L adrenaline results in the depolarization of resting membrane potential (RP) exclusively in PV_dis_ leading to intra-PV conduction dissociation. Two simultaneous microelectrode recordings from PV_dis_ (top recording in red) and PV_ost_ (bottom recording in blue) are shown. Below, selected time windows (gray rectangles) are shown enlarged before (baseline) and during PV_dis_ inexcitability under 50 nmol/L adrenaline. Left atria (LA) was constantly paced with S1S1 = 300 ms. **(B)** Application of 1 μmol/L adrenaline recovered intra-PV conduction suppressed by 50 nmol/L adrenaline. Below, selected time windows (gray rectangles) are shown enlarged before and after the recovery of excitability of PV_dis_.

Experimental protocol is shown schematically in Figure 1. Measurements were performed 40–60 min after the isolation procedure. Three adrenaline concentrations (25–100 nmol/L, 1 and 10 μmol/L) were tested at baseline (no stretch applied), under mild stretch (150 mg of weight applied, which approximately corresponds to a physiological pulmonary venous pressure of 1 mmHg calculated as applied weight × gravity constant/cross-section area of the PV preparation) and under moderate stretch (10 g of applied weight corresponding approximately to 26 mmHg of pulmonary venous pressure) as described previously (Egorov et al., 2019). For each condition, we first tested nanomolar concentrations of adrenaline (25–100 nmol/L) required to induce inexcitability within the PV_dis_ (determined as a failure of excitation propagation from PV_ost_ to PV_dis_ under constant LA pacing and APA in PV_dis_ < 20 mV, see Figure 2A). To test if PV_dis_ inexcitability was associated with adrenaline-induced RP depolarization rather than microelectrode stability, 1 μmol/L acetylcholine was briefly applied to stimulate the acetylcholine-activated outward potassium current *I*_K,ACh_, hyperpolarize the RP, and recover intra-PV conduction. After that, acetylcholine was washed out, and the next concentration of adrenaline was applied for 10 min. Preparations were constantly paced at stable S1S1 = 300 ms cycle length. To characterize spontaneous activity within the PV, electrical pacing was stopped for 5–10 min under each experimental condition. The protocol was repeated for 150 mg and 10 g of weight. Each weight was applied for an hour while adrenaline was washed out. The protocol was about 4–5 h long and applied to all the preparations tested. Importantly, in all experiments, PV preparations were functionally intact throughout the entire experiment as we demonstrated previously (Egorov et al., 2019) and tested here by their response to regular applications of acetylcholine.

Arrhythmia score was defined according to a type of spontaneous electrical activity event as follows: 0, no spontaneous activity; 1, an irregular sporadic electrical activity; 2, irregular bursts of fast electrical activity; 3, irregular bursts of fast electrical activity that was suppressed by atrial pacing; 4, a stable, but slow PV rhythm that could not be suppressed by atrial pacing; and 5, a fast regular PV rhythm that could not be suppressed by atrial pacing and was complicated by echo extra beats.

### Statistics

Student’s *t*-test was used in two-group comparisons. Multiple groups of normally distributed data of similar variance were compared by one- or two-way ANOVA. For multiple comparisons, the Bonferroni’s corrected *P* value is shown. All statistical analyses were performed using GraphPad Prism 5 or Origin version 6.1. A value of *P* < 0.05 was considered statistically significant. Values were presented as mean ± SEM.

## Results

Similar to what we have recently demonstrated (Egorov et al., 2019), mild stretch (150 mg) did not affect PV electrophysiology at baseline. Changes in RP, APA, and AP duration (APD) were not observed in both PV_ost_ and PV_dis_. In contrast, moderate (10 g) stretch significantly depolarized the RP specifically in PV_dis_ (-56 ± 2 mV vs. -82 ± 1 mV at baseline, *P* < 0.01) suppressing APA and resulting in inexcitability of PV_dis_ that was evident from a failure of the propagation of excitation toward PV_dis_ (Figure 1). In PV_ost_, neither RP or APA were changed at moderate stretch, while APD was significantly prolonged from 55 ± 2 ms to 84 ± 8 ms, respectively (*P* < 0.01).

**FIGURE 3:**
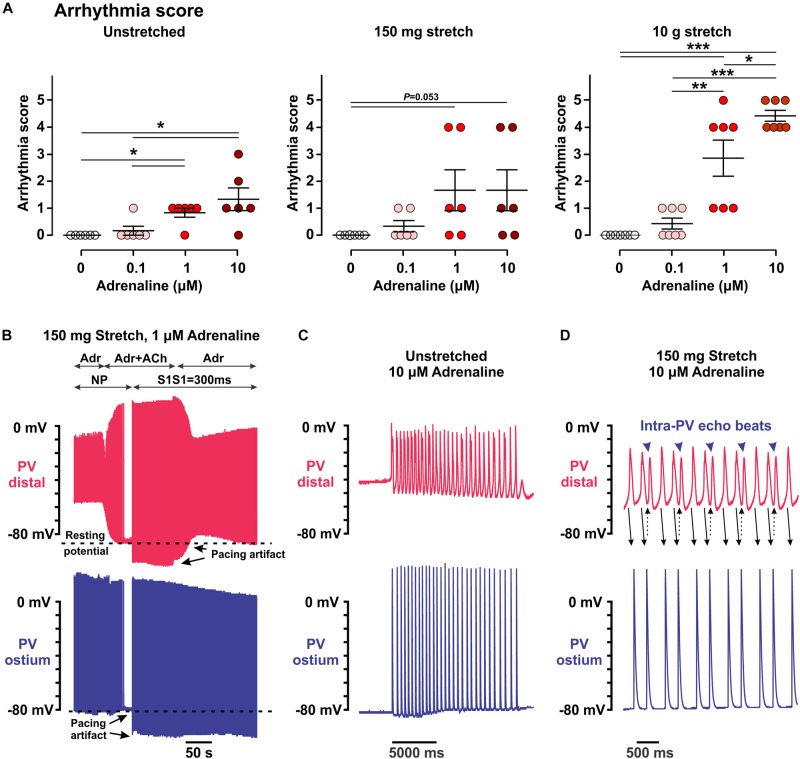
Arrhythmic events induced within the pulmonary vein (PV) myocardium by low (25–100 nmol/L) and high (1–10 μmol/L) concentrations of adrenaline in unstretched and stretched (150 mg and 10.5 g) preparations. **(A)** Arrhythmia score calculated as described in the section “Materials and Methods.” *N* = 6–7 rats. *P* values are calculated by repeated measurements two-way ANOVA with Bonferroni correction. **(B**–**D)** Representative examples of arrhythmic events recorded in PV preparations under different conditions. Two simultaneous microelectrode recordings from PV_dis_ (top recording in red) and PV_ost_ (bottom recording in blue) are shown for each event. **(B)** A brief application of 1 μmol/L acetylcholine (ACh) in 150 mg stretched preparation hyperpolarized the RP in PV_dis_ back to a baseline level and suppressed spontaneous PV activity (no pacing, NP) under 1 μmol/L adrenaline (Adr) application. After ACh washout, spontaneous slow PV rhythm (arrhythmia score is 3) was suppressed by atrial pacing (S1S1 = 300 ms). **(C,D)** Stable slow rhythm (arrhythmia score is 4, **C**) and fast regular rhythm (arrhythmia score is 5, **D**) induced by 10 μmol/L adrenaline in unstretched **(C)** and 150 mg stretched **(D)** PV preparations.

Low concentrations of adrenaline (25–100 nmol/L) depolarized the RP selectively within PV_dis_ (by 26 ± 2 mV at baseline and 18 ± 1 mV at mild stretch, *P* < 0.001) and did not change the RP in PV_ost_ measured during a stable atrial stimulation of S1S1 = 300 ms (Figure 1). This subsequently suppressed an APA in PV_dis_ and resulted in the development of intra-PV conduction dissociation (Figure 2A). At moderate stretch, this further depolarized the RP in the setting of PV_dis_ inexcitability by 5.9 ± 1.1 mV at 10 g, *P* < 0.01. A brief application of acetylcholine hyperpolarized the RP back to baseline values and successfully recovered PV_dis_ excitability in all preparations (Figure 3B).

Subsequent application of 1 μmol/L adrenaline recovered PV_dis_ excitability and the intra-PV conduction in all the preparations tested (see representative example in Figure 2B). In unstretched and 150 mg stretched preparations, it was associated with significant RP hyperpolarization and recovery of the APA (*P* < 0.01) in PV_dis_. In contrast, in 10 g stretched preparations, recovery of the intra-PV conduction was not associated with changes in the RP and only partial recovery of the APA in PV_dis_ (Figure 1). In PV_ost_, neither RP or APA were changed under 1 and 10 μmol/L adrenaline in both mildly and moderately stretched preparations.

Although 25–100 nmol/L adrenaline could strongly depolarize the RP in PV_dis_, this was not associated with the induction of automatic activity, both in unstretched and stretched preparations (arrhythmia index of 0.17 ± 0.17, *P* = 0.340 in unstretched preparations, 0.33 ± 0.21, *P* = 0.145, and 0.43 ± 0.20, *P* = 0.055, in 150 mg and 10 g stretched preparations, respectively; no spontaneous activity was observed without adrenaline; Figure 3A). This was opposite to higher concentrations of adrenaline (1–10 μmol/L), which evoked spontaneous activity in all preparations studied, with (unstretched and 150-mg stretched) and without (10-g stretched) concomitant hyperpolarization of the RP (Figures 3C,D). Moderate stretch significantly facilitated the triggering of rapid PV firing by adrenaline (arrhythmia index: 4.4 ± 0.2 vs. 1.3 ± 0.4 in unstretched, *P* < 0.001, and 1.7 ± 0.8 in mildly stretched preparations, *P* < 0.005, at 10 μmol/L adrenaline) and induced frequent episodes of intra-PV “echo” beats (Figure 3D) (Egorov et al., 2015; Bun et al., 2018).

**FIGURE 4:**
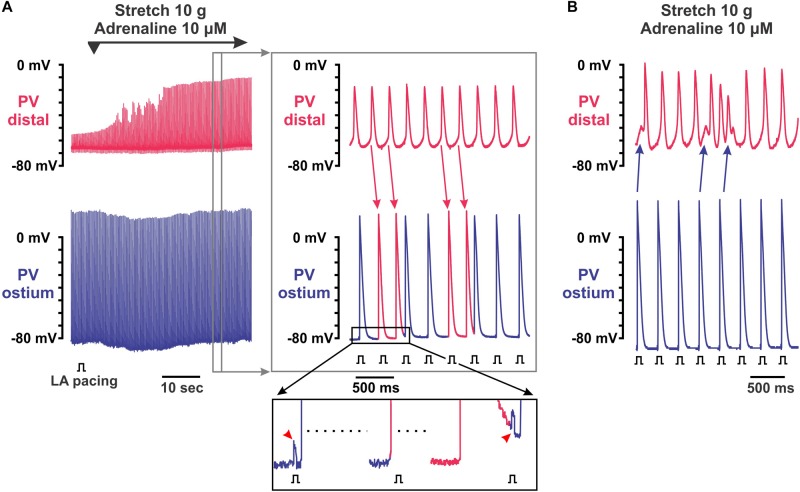
Complex electrical activity recorded in pulmonary vein (PV) preparations under 10 μmol/L adrenaline applied during 10 g stretch. Two simultaneous microelectrode recordings from PV distal (top recording in red) and PV ostium (bottom recording in blue) are shown. **(A)** Recovery of intra-PV conduction with arrhythmogenic activity after application of 10 μmol/L adrenaline in 10 g stretched PV. Left panel shows a continuous 1-min recording of adrenaline application, while right panel represents an enlarged section of the left recording indicated by a gray rectangle. Spontaneous activity was induced in distal PV and not suppressed by left atrial (LA) pacing. Instead, a faster rhythm in distal PV interfered with a slower rate of electrical pacing resulting in frequent atrial extra beats (shown as red action potentials in PV ostium recordings; red arrows indicate propagating PV distal beats). **(B)** Burst of fast electrical activity triggered in distal PV by atrial extra beat interfered with intrinsic PV rhythm.

Spontaneous activity induced by adrenaline in moderately stretched preparations were characterized by complex interactions between PV_dis_ and PV_ost_. Figure 4A shows an example of the initiation of PV spontaneous activity by application of 10 μmol/L adrenaline in a 10-g stretched preparation that led to irregular atrial extra beats. This activity was not suppressed by LA pacing. Instead, a faster rhythm in PV_dis_ interfered with a slower rate of electrical pacing resulting in frequent atrial extra beats (shown in Figure 4A as red APs in PV_ost_ recordings). Atrial extra beats triggered from PV_dis_ had different morphology as shown in the enlarged insert below: while paced APs (blue in PV_ost_ recording) followed pacing artifact, triggered extra beats (red in PV_ost_ recording) were not synchronized with atrial pacing and not associated with pacing artifacts. Another example shown in Figure 4B demonstrates how atrial beats can initiate arrhythmogenic burst of fast electrical activity in PV_dis_. Altogether, these findings show how spontaneous activity induced by sympathetic stimulation and facilitated by PV stretch can interfere with a stable atrial rhythm resulting in potentially arrhythmogenic atrial extra beats that can initiate atrial fibrillation.

## Discussion

Our findings demonstrate complex interactions between the sympathetic tone and mechanical stretch in the development of arrhythmogenic activity within PVs. First, we found a biphasic effect of adrenaline on PVs; while low concentrations of adrenaline (25–100 nmol/L) depolarized the RP and led to intra-PV conduction dissociation, high doses of adrenaline (1–10 μmol/L) led to RP hyperpolarization and the development of spontaneous electrical potentials within the PV. This biphasic effect of adrenaline could be linked to a dose-dependent activation of predominantly α- at low concentrations and both α- and β-adrenergic receptors at high concentrations (Furchgott, 1967). It was previously shown that stimulation of β-adrenergic receptors decreases the membrane stabilizing outward current *I*_K1_ (Braun et al., 1992) and enhances the volume-regulated inward chloride current *I*_Cl,swell_ (Ellershaw et al., 2002) that, together, would lead the RP depolarization as observed here and previously by Doisne et al. (2009). In contrast, stimulation of β-adrenergic receptors by high concentrations of adrenaline would increase *I*_K1_ but decrease *I*_Cl,swell_, hyperpolarizing the RP. Importantly, all these effects were observed under a stable atrial pacing when PV RP did not differ from that measured in the left ventricle as shown here (Figure 1) and previously (Doisne et al., 2009; Egorov et al., 2015).

We also found that pathological (moderate) stretch facilitated the development of arrhythmogenic ectopic activity induced by high concentrations of adrenaline in PVs. At depolarized RPs under stretch, application of adrenaline led to more regular and faster PV automaticity compared with nonstretched conditions (Figure 3A). Interestingly, although both moderate stretch and low concentrations of adrenaline significantly depolarized the RP, those were not associated with the induction of automatic activity and required an application of high concentrations of adrenaline. This indicate a crucial combination of pathologically depolarized RP and enhanced Ca^2+^ handling to form both a substrate [at cellular as well as tissue levels (Egorov et al., 2019)] and a trigger [in a form of early afterdepolarizations (Patterson et al., 2005)] for a stable spontaneous activity within PVs. As it was shown by Patterson et al. (2005), suppression of ryanodine receptor activity (by ryanodine) or reduction in the transmembrane gradient driving Na/Ca exchange (by increase [Ca^2+^]_o_ from 1.35 to 5 mM) completely suppressed spontaneous firing from PVs, without changed in RP.

Importantly, spontaneous electrical activity induced in the stretched PV by adrenaline was not suppressed by atrial pacing. Moreover, it led to frequent atrial extra beats that may potentially trigger arrhythmogenic atrial extra beats and thus initiate atrial fibrillation. It should be also noted that myocardium dilation during pressure and/or volume overload of the atria could result in heterogeneous distribution of wall stress creating regions of conduction slowing and thus facilitating the induction of atrial fibrillation by PV extra beats. Altogether, our findings highlight an arrhythmogenic impact of PV stretch in the development of atrial arrhythmias under elevated autonomic tone, which could play a critical role in patients with elevated blood pressure associated with hypertension, heart failure, and valvular disease.

## Data Availability Statement

The datasets generated for this study are available on request to the corresponding author.

## Ethics Statement

The animal study was reviewed and approved by the Animal Care and Use Committee of the Cardiology Research Center (Moscow, Russia).

## Author Contributions

YE, LR, and AG substantially contributed to the conception and design of the work; the acquisition, analysis, or interpretation of the data and literature; drafting of the work critically for important intellectual content; providing of approval for publication of the content; and agreeing to be accountable for all aspects of the work in ensuring that questions related to the accuracy or integrity of any part of the work are appropriately investigated and resolved.

## Conflict of Interest

The authors declare that the research was conducted in the absence of any commercial or financial relationships that could be construed as a potential conflict of interest.
